# Mediastinal hematoma after trans-radial cerebral angiography: a case report

**DOI:** 10.1186/s12883-024-03714-z

**Published:** 2024-07-03

**Authors:** Peipei Ma, Zhenyu Gong, Meng Du, Deyuan Zhu, Peng Li, Yibin Fang

**Affiliations:** 1 Department of Neurovascular Disease, Shanghai Fourth People’s Hospital, Tongji University, 1279 Sanmen Road, Shanghai, 200434 China; 2 Department of Neurosurgery, Klinikum rechts der Isar, Technical University of Munich, Munich, Germany

**Keywords:** Mediastinal hematoma, Digital subtraction angiography, Trans-radial access, Neurointervention, Internal carotid arteries, Case report

## Abstract

**Background:**

Trans-radial (TRA) access has become increasingly prevalent in neurointervention. Nonetheless, mediastinal hematoma after TRA is an infrequent yet grave complication associated with a notably elevated mortality rate. While our review found no reported mediastinal hematoma cases managed conservatively within neuro-interventional literature, similar complications are documented in cardiac and vascular interventional radiology, indicating its potential occurrence across disciplines.

**Case Presentation:**

Carotid computed tomography angiography (CTA) showed calcified plaques with stenosis (Left: Severe, Right: Moderate) in the bilateral internal carotid arteries (ICAs) of an 81-year-old male presented with paroxysmal weakness in the right upper limb. Dual antiplatelet therapy with aspirin and clopidogrel was administered. On day 7, DSA of the bilateral ICAs was performed via TRA. Post-DSA, the patient experienced transient loss of consciousness, chest tightness, and other symptoms without ECG or MRI abnormalities. Hemoglobin level decreased from 110 g/L to 92 g/L. Iodinated contrast-induced laryngeal edema was suspected, and the patient was treated with intravenous methylprednisolone. Neck CT indicated a possible mediastinal hemorrhage, which chest CTA confirmed. The patient’s treatment plan involved discontinuing antiplatelet medication as a precautionary measure against the potential occurrence of an ischemic stroke instead of the utilization of a covered stent graft and surgical intervention. Serial CTs revealed hematoma absorption. Discharge CT showed a reduced hematoma volume of 35 × 45 mm.

**Conclusions:**

This case underscores the need for timely identification and precise manipulation of guidewires and guide-catheters through trans-radial access. The critical components of successful neuro-interventional techniques include timely examination, rapid identification, proper therapy, and diligent monitoring.

## Background

In 1997, Cowling et al. first reported a case of carotid angiography performed using trans-radial access [1]. Over the past few decades, trends in neuro-interventional therapies, particularly digital subtraction angiography (DSA) and carotid artery stenting (CAS) via trans-radial access (TRA), have become increasingly apparent. Compared to transfemoral access (TFA), TRA has gained popularity because of its lower risk of bleeding, fewer vascular complications, earlier mobilization, and enhanced patient comfort [2–4]. However, it is noteworthy that TRA has been associated with a slightly higher risk of periprocedural stroke compared to transfemoral access. A study by Kuhn et al. highlights this concern, providing essential insights for neurointerventionalists in selecting the optimal access route [5]. Other common complications of trans-radial access include radial artery occlusion, puncture site hemorrhage, and radial artery spasms [6, 7]. Mediastinal hematoma is a rare but severe complication of neuro-interventional procedures involving trans-radial access. Excessive manipulation leading to vascular perforation can occur when guidewires or catheters pass through the brachial, subclavian, and brachiocephalic arteries. Such complications typically occur when the guidewire or catheter shears the arterial wall in the annular, tortuous, or small-caliber arterial segments. In this report, we present a rare case of mediastinal hematoma causing chest tightness. The specific cause of the mediastinal hematoma remains unclear, but it is suspected to be a subclavian artery puncture injury during a DSA examination.

## Case presentation

An 81-year-old male was admitted on April 21, 2023, for a one-month history of paroxysmal right upper limb weakness. The patient denied any history of trauma, cardiovascular disease, smoking, or alcohol consumption. Post-admission carotid computed tomography angiography (CTA) revealed calcified plaques in the initial segments of the bilateral internal carotid arteries (ICAs) accompanied by stenosis, with severe stenosis in the left ICA and moderate stenosis in the right ICA. Given the patient’s symptoms of right upper limb weakness, the severe stenosis in the left ICA is considered symptomatic. Dual antiplatelet therapy comprising aspirin (100 mg; Bayer, Leverkusen, Germany), clopidogrel (75 mg; Sanofi, Paris, France), and other standard treatments were initiated.

**Fig. 1:**
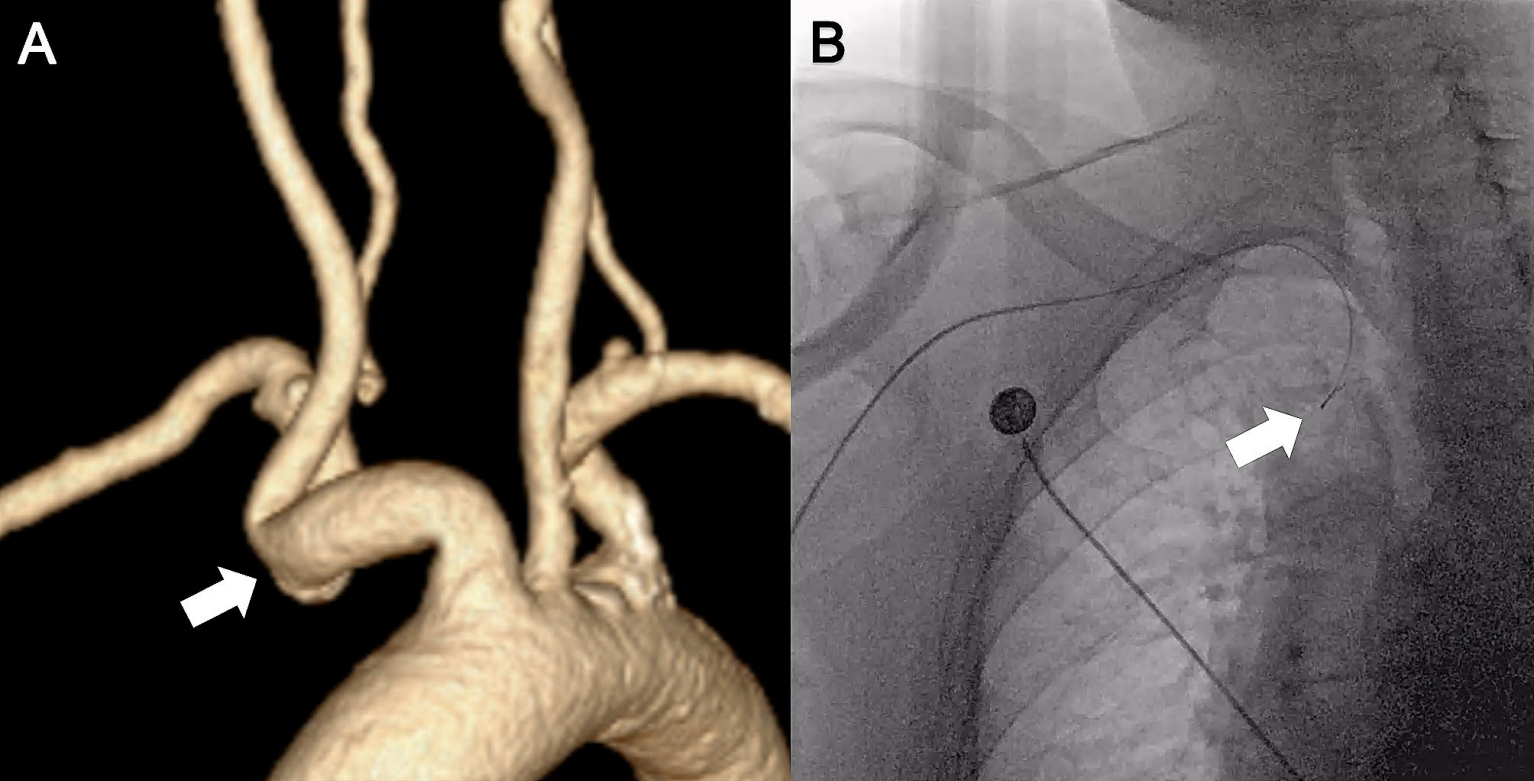
Demonstration of tortuous subclavian artery, making it difficult for the guide wire to pass through. (**A**) Computed tomography angiography. (**B**) Trans radial cerebral angiography

On the 7th day of hospitalization, DSA was performed under local anesthesia via radial artery access to assess the carotid artery condition further. The operator involved in this case is part of our neurointerventional team at a center renowned for emphasizing trans-radial access. With over three years of independent experience in trans-radial angiography and treatment, the operator has completed over 2000 cases. The right radial artery was chosen for puncture, and the Seldinger technique was employed. After a successful puncture of the right radial artery, a 5 F radial sheath was inserted. A 5 F Simmons-2 glidecath catheter (Int Medical, China) was advanced under the guidance of 0.035-inch stiff guidewire (Terumo, Tokyo, Japan). Maneuvering is highly challenging because of the anomalous tortuosity of the right subclavian artery, requiring multiple attempts to traverse the tortuous segment under the guidance of the Terumo guidewire (Fig. 1). Bilateral angiography of the common carotid and subclavian arteries was performed. According to the North American Symptomatic Carotid Endarterectomy Trial (NASCET) criteria [8], severe stenosis quantified as 90.6% was observed at the origin of the left ICA, moderate stenosis quantified as 55.4%, along with lesions, were observed in the initial segment of the right ICA. The whole fluoroscopic time for this case was 40 min. The catheter was removed, and hemostasis was achieved at the puncture site. The patient safely returned to the ward with a plan for elective left ICA balloon angioplasty and stent placement.

Twenty minutes after the procedure, the patient suddenly reported chest tightness, followed by a transient loss of consciousness that resolved within seconds. Subsequent symptoms included dizziness, neck discomfort, persistent chest tightness, nausea, and vomiting of gastric contents. No fever or chills were noted, and the vital signs remained stable. No myocardial infarctions or ischemic changes were observed on electrocardiography. Brain MRI showed no new cerebral infarcts. Hematological and biochemical tests revealed decreased hemoglobin levels from 110 g/L to 92 g/L but no other significant abnormalities. Symptomatic antiemetics and supplemental oxygen treatment were provided, leading to symptom improvement.

In the early hours of the same day postoperatively, the patient developed symptoms of dyspnea and dysphagia, although other vital signs remained stable. Iodinated contrast agent-induced laryngeal edema was suspected, and intravenous methylprednisolone sodium succinate 40 mg was rapidly administered. Subsequent symptom improvement was observed. A laryngoscopy revealed no abnormalities. Subsequent neck CT tomography suggested disorganizing the structures in the upper mediastinum, raising the possibility of a hemorrhage. Chest CT without contrast confirmed a mediastinal hematoma measuring approximately 69 × 62 mm. Chest CTA angiography revealed no significant aortic abnormality, contrast extravasation, or increased mediastinal bleeding volume. Upon reviewing the surgical procedure, no clear contrast agent extravasation was observed following ROADMAP imaging conducted in the right subclavian artery. Therefore, we speculate that repeated super-selection of the subclavian artery using the guidewire caused a minor injury to the vessel, although this was unnoticeable during the operation. Temporarily discontinuing antiplatelet therapy was decided because of the patient’s symptomatic carotid artery stenosis and ongoing antiplatelet treatment with aspirin and clopidogrel. However, considering the high risk of ischemic stroke because of vascular pathology in the neck, it was not necessary to initiate any hemostatic agents. Subsequently, the patient’s vital signs were closely monitored, and hemoglobin levels were continuously assessed dynamically. Serial chest CT scans were performed to monitor the mediastinal hematoma. Elective left ICA balloon angioplasty and stent placement were postponed until significant improvement in the mediastinal hematoma was observed (2 months later). This decision underscores the necessity of adapting interventional strategies based on the patient’s evolving clinical status, especially when managing complications such as hematoma formation. The resumption of dual antiplatelet therapy was carefully timed following a dynamic reassessment of the hematoma through follow-up chest CT scans, ensuring the hematoma had sufficiently resolved before proceeding with further vascular interventions. .

**Fig. 2:**
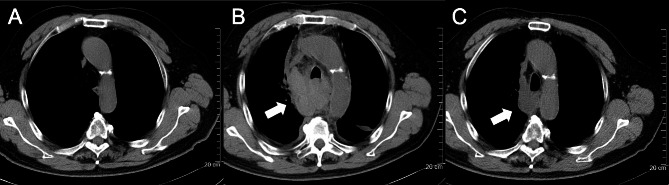
Chest computed tomography. (**A**) The pre-DSA image shows the normal mediastinum, trachea, and lung range. (**B**) The post-DSA image shows mediastinal hematoma and tracheal compression. (**C**) The image at discharge (after 48 days) shows decreased mediastinal hematoma and increased tracheal diameter

Serial CT during the hospital stay revealed gradual absorption of the mediastinal hematoma. Follow-up chest CT at discharge revealed a significantly reduced mediastinal hematoma, measuring 35 × 45 mm (Fig. 2).

## Discussion

A review of the literature, particularly from the cardiovascular field, reveals that mediastinal hematoma as a complication of the trans-radial procedure, though rare, is not unprecedented. Studies such as those by Jao et al. and Wang et al. have documented similar complications, underscoring the potential for significant vascular trauma even with the most meticulous catheter and guidewire manipulation [9, 10]. These reports highlight the clinical spectrum of hematoma development following trans-radial access, ranging from localized hematomas to extensive vascular injuries necessitating emergency interventions. The cardiovascular literature, therefore, offers valuable insights into the mechanisms, risk factors, and preventive strategies directly applicable to neuro-interventional procedures. This cross-disciplinary learning emphasizes the importance of vigilance and precision in catheter and guidewire manipulation and the adoption of best practices from cardiovascular interventions to minimize complications in neuro-interventional settings. The occurrence of mediastinal hematoma in this patient underscores the technical difficulties inherent in the trans-radial DSA procedure [11–13]. Similar to findings in cardiovascular interventions, vascular tortuosity significantly challenges catheter and guidewire navigation, potentially leading to arterial damage and subsequent hematoma formation.

The development of a mediastinal hematoma following trans-radial cerebral angiography, as observed in our case, underscores the intricate interplay between anatomical challenges and procedural techniques. Notably, the vascular tortuosity of the subclavian artery, causing considerable difficulty in catheter and guidewire advancement. It is plausible that this manipulation results in arterial damage and subsequent hematoma formation. Surgeons should remain acutely aware of the risks associated with navigation through tortuous arterial segments because such conditions often necessitate forceful manipulation, which increases the risk of complications [14]. This condition not only necessitates precise navigation but also increases the likelihood of complications such as vascular perforation Reflecting upon similar occurrences in the cardiovascular field, it becomes evident that the mechanism of hematoma formation often involves the combination of mechanical stress on vulnerable vessels and the exacerbating factor of antithrombotic therapy. In particular, the act of navigating through tortuous vascular paths can induce shear stress and microtraumas, which, in the presence of antiplatelet medication, may culminate in significant bleeding and hematoma formation. Thus, understanding the potential causes and mechanisms of hematoma development is crucial for devising strategies to minimize such risks in future interventions.

Vascular difficulties may arise when guide catheters are maneuvered through tortuous arteries despite a properly positioned guidewire in the ascending aorta. These complications may occur because of applying forward pressure against the resistance. In the event of resistance, fluoroscopy should be employed to verify the relative placement of the distal guidewire and guide catheter. It is crucial to exercise caution and guarantee proper positioning of the guidewire before advancing the guide further. Several proven techniques, including balloon-assisted tracking, deep inhalation, and arm abduction, could be employed to mitigate the occurrence of vascular shearing resulting from the “razor effect” at the distal tip of the guide catheter [15]. Hydrophilic guide catheters can also be used to traverse complex anatomical structures. Rigid guide catheters are larger, have a more tortuous or curved form at the distal tip, and are more prone to trauma during catheter administration. Following this, the use of hydrophilic guidewires presents a distinct advantage due to their enhanced lubricity, reducing friction significantly and facilitating maneuvering through tortuous segments. However, the very feature that enables their smooth passage also increases the likelihood of inadvertent entry into small side branches, potentially leading to vessel damage. Thus, while hydrophilic wires are beneficial for navigating complex paths, they require vigilant operator control to ensure safety, particularly keeping the wire’s distal tip within the operator’s field of view to mitigate the risk of subintimal dissection and perforation [16]. Additionally, in this case, the hematoma was considered to have been caused by guidewire injury during repeated super-selection of the tortuous subclavian artery. This finding suggests that when performing interventional procedures via the radial artery approach, for vessels that are tortuous and difficult to super-select, we should consider using a “J” shaped guidewire for guidance to avoid or minimize injury to the vessel by the tip of the guidewire during the super-selection process. In reflecting on the procedural aspects of this case, it is noteworthy that the fluoroscopic time amounted to 40 min, highlighting the intricate navigation required through the patient’s vascular system. The complexity of maneuvering the guidewire and catheter through tortuous vessels underscored the necessity for meticulous planning and execution. Despite the procedural challenges, the onset of mediastinal hematoma symptoms occurred post-procedure once the patient had returned to the ward rather than during the angiographic process itself. This temporal gap between the procedure and symptom onset emphasizes the importance of vigilant post-procedural monitoring. It is also noteworthy that roadmap guidance was utilized in this instance, reflecting our commitment to employing best practices in procedural navigation. However, the occurrence of a mediastinal hematoma post-procedure highlights the unpredictable nature of neurointerventional surgeries and the need for constant vigilance, even when using roadmap guidance. This experience emphasizes that while roadmap guidance can significantly reduce the risk of aggressive wire manipulation and unintended vessel injury, it cannot entirely eliminate the inherent risks of navigating tortuous anatomy. Further, our experience suggests that when faced with difficult navigation through tortuous vasculature, allocating additional time to thoroughly understand the vascular anatomy before attempting passage, coupled with a retrospective angiographic review of navigated segments, may serve as critical steps in mitigating the risk of complications such as mediastinal hematoma. Such practices not only enhance procedural safety but also contribute to the broader neurointerventional community’s understanding of managing and potentially preventing complications associated with complex vascular navigation.

The patient, in this case, was administered oral antiplatelet medication upon admission, which could have potentially contributed to the onset of the mediastinal hematoma. Antiplatelet agents are widely known for their role in inhibiting platelet aggregation, which increases the risk of bleeding. Additionally, spontaneous mediastinal hematomas have been reported in patients undergoing anticoagulant therapy [17, 18]. Based on the pharmacological effects of these drugs, it is plausible that administering antiplatelet treatment may increase a patient’s vulnerability to hematoma formation after DSA. While these agents are indispensable for preventing thromboembolic events, their use increases the bleeding risk, as highlighted by our case. This paradox underscores the need for a balanced approach to antithrombotic therapy, considering the individual patient’s risk profile and the specific procedural context.

Finally, this case highlights the importance of maintaining a broad differential diagnosis when patients exhibit respiratory distress after interventional procedures. An allergic reaction to the iodinated contrast agent was initially suspected, prompting a laryngoscopic examination, which yielded no positive findings [19, 20]. The diagnosis of a mediastinal hematoma was confirmed using computed tomography. Prompt identification and treatment are critical for successful management of life-threatening complications. This case reinforces the importance of remaining vigilant for uncommon but severe complications, especially when initial diagnostic avenues do not yield conclusive results.

## Conclusions

In summary, vascular perforations from advancing guide catheters via the TRA are rare. During interventional procedures, neurosurgeons should use caution and refrain from forcefully advancing the guide catheter and wire in the presence of resistance without fluoroscopy. The effective treatment of fatal complications requires early assessment, timely identification, proper therapy, and constant monitoring.

## Data Availability

No datasets were generated or analysed during the current study.

## Abbreviations

DSA: Digital subtraction angiography
CTA: Computed tomography angiography
TRA: Trans-radial arterial
CAS: Carotid artery stenting
ICA: Internal carotid artery
